# Sulfate depletion triggers overproduction of phospholipids and the release of outer membrane vesicles by *Neisseria meningitidis*

**DOI:** 10.1038/s41598-019-41233-x

**Published:** 2019-03-18

**Authors:** Matthias J. H. Gerritzen, Dirk E. Martens, Joost P. Uittenbogaard, René H. Wijffels, Michiel Stork

**Affiliations:** 1 Institute for Translational Vaccinology (Intravacc), Process Development Bacterial Vaccines, P.O. Box 450, 3720 AL Bilthoven, The Netherlands; 2 Wageningen University, Bioprocess Engineering, P.O. Box 16, 6700 AA Wageningen, The Netherlands; 3 Institute for Translational Vaccinology (Intravacc), Analysis, Delivery, and Formulation, P.O. Box 450, 3720 AL Bilthoven, The Netherlands; 4 Nord University, Faculty of Biosciences and Aquaculture, P.O. Box 1409, 8049 Bodø, Norway

## Abstract

Outer membrane vesicles (OMVs) produced by bacteria are interesting vaccine candidates. OMVs are nanoparticles that contain many immunogenic components, are self-adjuvating, and non-replicative. Despite recent insights in the biogenesis of OMVs, there is no consensus on a conserved mechanism of OMV release and the OMV yield from bacterial cultures remains low. For *Neisseria meningitidis*, a Gram-negative human pathogen causing meningitis and sepsis, a feasible OMV production method based on triggering OMV release by cysteine depletion has been described. In this study, we investigated the mechanism behind this external trigger for OMV release to improve the production process. Since enhanced OMV release upon cysteine depletion was associated with oxidative stress and redox responses, we investigate the influence of more oxidized sulfur sources on OMV release. We show that *N. meningitidis* grows similarly on sulfate, the most oxidized sulfur source, and OMV release is triggered by sulfur depletion in general. Sulfate depletion induced increased release of OMVs over cysteine depletion. Proteomics showed that sulfur depletion resulted in oxidative stress responses and upregulated phospholipid and LPS biosynthesis. Furthermore, OMVs produced by sulfur depletion were enriched in phospholipids. Mechanistically, we hypothesize that sulfur depletion results in overproduction of phospholipids causing increased bulging of the outer membrane and subsequent OMV release.

## Introduction

Outer membrane vesicles (OMVs) are spherical nanoparticles that contain the natural components of the bacterial outer membrane, including antigenic components^1^. OMVs have been effectively used as vaccines^2–6^. The OMV-based vaccines are currently not based on OMVs that are spontaneously released by the bacteria (sOMVs), but on vesicles extracted by a detergent (dOMVs). This extraction method was required to remove endotoxic lipopolysaccharides (LPS) from the OMVs, to make them suitable as vaccine. However, detergent extraction also caused a reduction in lipoproteins, reduced vesicle stability, and increased amounts of cytoplasmic components, all of which lower the vaccine quality^7^. Recently, LPS has been genetically engineered to reduce the toxicity of LPS by altering the LPS structure^8,9^. This approach of genetic LPS detoxification makes the detergent extraction obsolete and allows much milder detergent-free extraction methods. These milder OMV extraction methods can be used to produce detergent-free extracted OMVs (eOMVs) with improved yield, stability and immunogenicity as compared to dOMVs^10,11^. Alternatively, genetic detoxification of LPS also allows the use of sOMVs as vaccines.

Using sOMV over eOMVs simplifies the vesicle production process. OMV extraction requires several additional unit operations while sOMVs could be directly obtained from the culture supernatant. Another advantage of using spontaneous vesicles as vaccines is the enhanced immunogenicity of the vesicles^10^. Compared to both eOMVs and dOMVs, the immune response against sOMVs from *Neisseria meningitidis* (Nm) is higher and shows a broader cross-protection against PorA types that are not included in the vesicles itself^10^. sOMV production however, is challenging since typical sOMV productivity is low. The release of OMVs has been proposed as a regulated mechanism based on the observation that OMVs differ in protein composition from the bacterial outer membrane^12–16^. Many biological functions have been ascribed to OMVs, that are advantageous for the survival chances of the bacterium by for example delivery of virulence factors or modulation of host immune response^1,17^. Progression in understanding OMV biogenesis has been made, however the mechanism of OMV formation remains unclear and even the question remains whether a conserved mechanism exists^7,18^. The biogenesis of OMVs has been categorized in three models^19^. In a first model, OMV release is based on the maintenance of lipid asymmetry in the outer membrane, that is based on increased phospholipid content in the outer leaflet of the outer membrane instead of LPS. A second model proposes that OMV release is induced by misfolded and unfolded proteins in the periplasm. In a third model, OMV formation is induced by alterations in the stability of the outer membrane due to LPS modifications.

Since the exact biogenesis of OMVs remains unknown, engineering approaches were applied to improve OMV productivity. One approach is based on the observation of increased vesicle release by strains with a reduced linkage of the outer membrane to the peptidoglycan^1^. It has been shown for several bacterial species that reducing the outer membrane (OM)-peptidoglycan linkage, by knockout mutation of membrane anchoring proteins, increases the release of OMVs^10,20–23^. In this work, we use the knockout of the membrane anchoring RmpM protein to increase the sOMV release of Nm. Additionally, we showed previously that cysteine depletion could be used as external trigger of Nm OMV release in a batch culture^24^. Cysteine depletion resulted in impaired sulfur supply, the onset of the stationary growth phase, oxidative stress as shown by upregulation of genes involved in oxidative stress responses, and in an increased OMV release. However, the mechanism of this increased vesicle formation remains unknown.

Based on the fact that oxidative stress responses seem to be involved in OMV release, we hypothesize that the oxidation state of the sulfur source affects the release of OMVs. Sulfur is an essential component of proteins and the sulfur metabolism of Nm has been extensively studied^25–28^. Originally it was described that reduced sulfur (cysteine or cystine) is necessary for growth^25,26^, and Catlin described an absolute requirement of cysteine for growth of a few Nm strains^29^. However, growth of *Neisseria* on the oxidized sulfur source sulfate has been shown^27,28^, indicating that Nm metabolism is capable of sulfate reduction. The cysteine biosynthesis in *Neisseria* species has been recently reviewed^30^. For the assimilation of cysteine from sulfate, sulfate is converted to adenosine 5′-phosphosulfate (APS) and via phosphoadenosine-5′-phosphosulfate (PAPS) the sulfur is reduced to sulfite^31^. After further reduction of sulfite to hydrogen sulfide the sulfur is incorporated in cysteine. It has been proposed that the Nm pathway may differ slightly from the described classical cysteine assimilatory pathway, since APS could be directly reduced to sulfite without PAPS as intermediate^31^. Besides sulfate, thiosulfate can be used as well for the assimilation of sulfur source since the presence of thiosulfate reductase has been shown^28^. Growth of Nm on oxidized sulfur sources is thus possible and the availability of multiple sulfur-acquisition routes illustrates the importance of sulfur in the metabolism of meningococci.

This study aims to increase the understanding of OMV formation in Nm triggered by sulfur depletion by looking at the influence of different sulfur sources on OMV release upon sulfur depletion. First, we assess growth of Nm on sulfate as the sole sulfur source in a chemically defined medium. Next, the effect of cysteine and sulfate depletion is assessed by proteomics. Lastly, to gain further insight in the mechanism of OMV biogenesis upon sulfur-source depletion, the biochemical composition of OMVs produced in different growth phases and upon both cysteine and sulfate depletion was assessed.

## Results

### Influence of sulfur depletion on sOMV release

**Figure 1:**
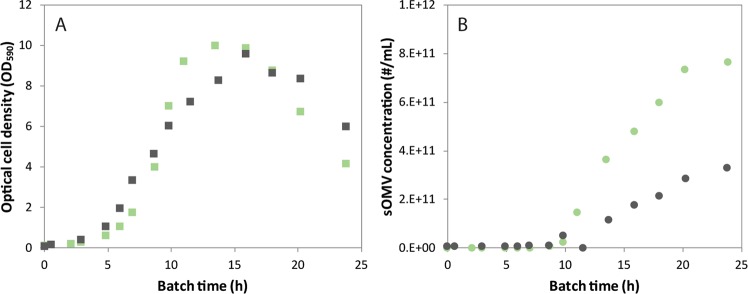
Growth and sOMV release of Nm during *s*ulfur source depletion. Nm cultures are grown on cysteine (black) and sulfate (green) as sulfur source. The growth curve based on optical density is shown in graph (**A**), and the concentration of OMV is given in graph (**B**). Graphs are the overlay of two replicate cultures to practically allow for sufficient data points covering 24 h.

After initial adaptation of Nm on sulfate, growth on other oxidized sulfur sources such as sulfite and thiosulfate was also possible without the requirement of an additional adaptation phase. Growth in shaker flasks on oxidized sulfur sources was comparable to growth on cysteine (Data not shown). Growth on sulfate, the most oxidized sulfur source was compared to growth on the preferred (reduced) sulfur source cysteine in benchtop bioreactor cultures. The dissolved oxygen tension of the cultures was controlled at 30% air saturation. The growth pattern of Nm on sulfate shows exponential growth with an average specific growth rate of 0.47 h^−1^ until the maximum optical density in this medium is reached (Fig. 1A). The average specific growth rate on cysteine is similar, 0.46 h^−1^. The carbon dioxide production pattern of the cultivation on both sulfur sources shows the typical pattern of a batch culture, with a respiratory quotient of 1.0 throughout the culture. Previously it was shown that cysteine depletion causes growth arrest^24^ and with similar biomass yields on sulfate we assume sulfate limitation is the cause of the onset of the stationary phase of the sulfate based culture. At this point in both cultures, other nutrients like the carbon, nitrogen, and phosphate source are still available in abundance (data not shown). Both cultures show minimal OMV release during the exponential growth phase (Fig. 1B). In the stationary phase both cultures produce sOMVs. However, the sOMV release upon sulfate depletion showed higher than during cysteine depletion.

### Proteome analysis of cysteine and sulfate depleted batch cultures

**Figure 2:**
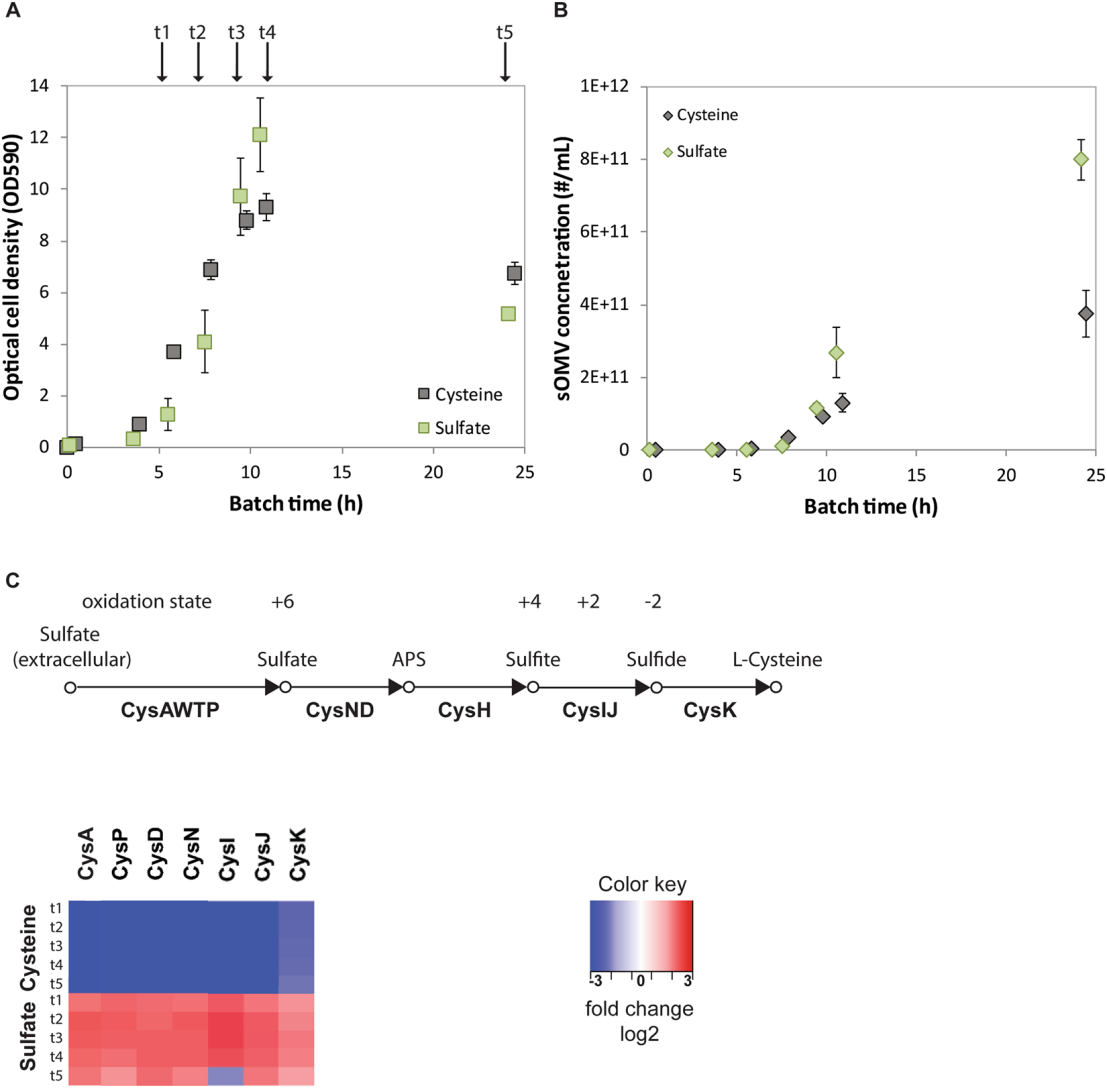
Experimental approach for proteomics. Triplicate Nm batch cultures on cysteine or sulfate as the sole sulfur source were sampled during exponential growth (t1, t2), onset stationary phase (t3, t4), and late stationary phase (t5) (**A**). The OMV concentration was measured by NTA throughout the cultivation (**B**). Error bars indicate standard deviation from the mean. The proteome is assessed for the cysteine assimilation pathway as shown in the heat map (**C**).

To understand how the bacterium copes with the onset of sulfur depletion during cultivation, the proteome was examined. Triplicate bioreactor cultures with either cysteine or sulfate as sole sulfur source were analyzed at different time-points during the cultivation (Fig. 2A). Culture supernatants showed depletion of the corresponding sulfur source at 7 h (Fig. 2A, time-point t2). Sulfur depletion results in increased OMV concentrations in the culture (Fig. 2B), confirming the previous results (Fig. 1). During growth in the presence of cysteine (t1), the proteome of Nm shows no upregulation of the cysteine biosynthesis pathway as expected as cysteine is sufficiently available (Fig. 2C, Supplemental File 1 for the complete dataset). The pathway is also not upregulated when cysteine becomes limiting (t3-t5), possibly because no sulfur is available for the generation of new proteins, or since no other sulfur source is available. CysW, CysT and CysH were below the detection limit of the LC-MS/MS analysis. During growth on sulfate the cysteine biosynthesis pathway was upregulated throughout the cultivation as expected.

**Figure 3:**
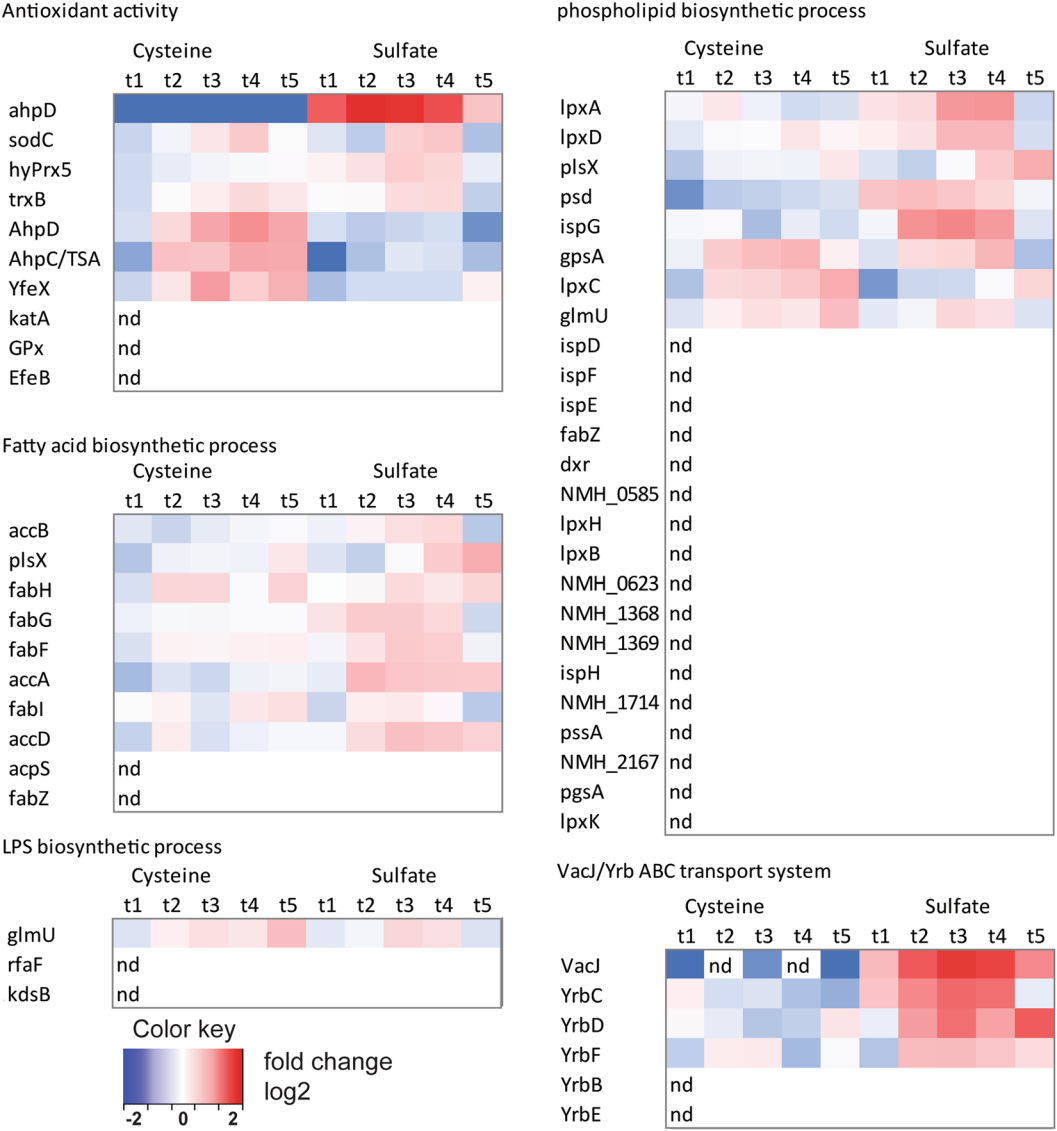
Heat maps based on gene ontology. Heat maps for specific gene ontology groups from the proteome dataset. The gene ontologies for antioxidant activity (GO:16209), Fatty acid biosynthesis (GO:6633), LPS biosynthesis (GO:9103), and phospholipid biosynthesis (GO:8654) are shown. Additionally, the expression level is shown of the VacJ/Yrb ABC transport system. VacJ was included although it was only detected in 8 out of 10 time-points, since the VacJ levels were near the detection limit in the cysteine culture. Samples indicated by “nd” were not detected.

We then performed gene ontology enrichment on the proteome data. Proteins categorized in the gene ontology antioxidant activity (GO:16209) were found to be upregulated upon cysteine depletion (time points t3, t4) (Fig. 3). This agrees to the previously described antioxidant and redox-stress responses found in the transcriptome upon cysteine depletion^24^. Upon sulfate depletion proteins with antioxidant activity are upregulated as well. Upon cysteine depletion, AhpD and AhpC are upregulated. Upon sulfate depletion these appear not to be regulated, while another protein with ascribed alkylhydroxyperoxidase activity (ahpD, E6MUC5) was observed to be regulated on sulfate as sulfur source. Proteins involved in phospholipid (GO:8654), fatty acid and LPS production (GO:9103) were upregulated after both cysteine depletion and sulfate depletion, simultaneously with increased OMV release. Both phospholipid biosynthesis and fatty acid biosynthesis proteins were more upregulated after sulfate depletion than after cysteine depletion. This upregulation is consistent with the increased release of OMV observed after sulfate depletion. Interestingly, the VacJ/Yrb ABC (ATP-binding cassette) transporter, which has a proposed role in maintaining lipid asymmetry in the outer membrane by transport of phospholipids from the outer leaflet to the inner leaflet, is strongly upregulated upon growth on sulfate as sulfur source, and is not regulated upon cysteine depletion.

### Sulfur depletion results in phospholipid enriched OMVs

**Figure 4:**
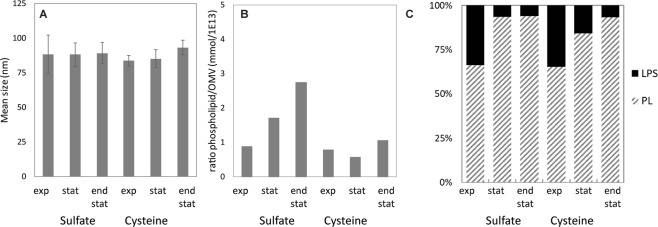
OMV characteristics upon sulfur depletion. OMV size by Nanoparticle Tracking Analysis of purified sOMV at different growth stages of the batch cultures on cysteine and sulfate (**A**). Error bars indicate standard deviation of the mean of 10 measurements. The biochemical composition of OMVs produced upon sulfur depletion show increased phospholipid/OMV ratio (**B**). The fatty acid distribution between phospholipid and LPS is shown upon sulfur source depletion (**C**).

During OMV production as a result of sulfur depletion, increased phospholipid production takes place, which could result in OMVs enriched in phospholipid content. This changed composition of the OMVs could also affect their size. To test this, OMVs were isolated from batch cultures containing either sulfate or cysteine, during the exponential growth phase, onset of the stationary phase and in the stationary phase. The size of OMVs was measured by NTA and was found to be similar throughout the cultivation and between sulfate depletion and cysteine depletion (Fig. 4A). A small trend in increased OMV size upon sulfur depletion was observed, although non-significant. The phospholipid content of OMVs increased upon sulfate depletion, while upon cysteine depletion the phospholipid content only shows a minor increase (Fig. 4B). The relative content of LPS, based on fatty acid analysis, decreased similarly for both sulfate and cysteine depletion (Fig. 4C).

## Discussion

This study shows that not cysteine depletion specific, but sulfur depletion in general induces OMV release in Nm cultures. Growth of Nm on sulfate was comparable to growth on cysteine as sulfur source. Upon sulfur depletion, the proteome showed increases in antioxidant activity, phospholipid biosynthesis and LPS biosynthesis. OMVs released after sulfur depletion were enriched in phospholipids. The most interesting finding was that sulfate depleted cultures showed increased OMV release over cysteine depleted cultures.

OMV formation upon sulfur depletion can be the result of increased phospholipid biosynthesis. Especially OMVs produced after sulfate depletion showed to be enriched in phospholipids. This finding was supported by the increased phospholipid biosynthesis observed in the proteome. Recently, an important role was shown for the VacJ/Yrb ABC (ATP-binding cassette) transporter in OMV biogenesis^16^. This transporter has a proposed role in maintaining lipid asymmetry in the outer membranes as phospholipid transporter^32^, by transporting phospholipids from the outer leaflet of the membrane to the inner leaflet, and is annotated in the Nm genome. It was shown by Roier *et al*. that the VacJ/Yrb ABC transporter was downregulated by the ferric uptake regulator (FUR) upon iron limitation in *H. influenzae, V. cholerae and E. coli*^16^. Disruptions of the VacJ/Yrb ABC transport system resulted in increased OMV formation, due to phospholipid accumulation in the outer membrane. This increase in OMV production in *H. influenzae* was not accompanied with increased fatty acid biosynthesis. In this study we show that sulfur depletion causes increased release of OMVs that are enriched in phospholipids. It should be noted that we used a strain with reduced linkage between the peptidoglycan and the outer membrane (*rmpM* knockout mutant). Interestingly, our Nm proteomics data upon sOMV formation by sulfate depletion showed simultaneous upregulation of the VacJ/Yrb ABC transporter and phospholipid biosynthesis. Possibly, this transporter is upregulated to counteract the accumulation of phospholipids in the outer membrane due to the increased synthesis and stop of growth. The overproduction of OMVs could be a method to dispose of the excess phospholipids in the outer membrane because the transporter has insufficient capacity. This suggests that upon sulfur depletion, OMV release is triggered in a different manner than downregulation of the VacJ/Yrb ABC transport system. Although it does support the OMV biogenesis model of phospholipid accumulation based OMV release^19^.

The increased phospholipid production upon growth on sulfate can be a result of the altered redox state. During cysteine assimilation from sulfate, NADPH is required to reduce the oxidized sulfur source^30^. NADPH replenishment is thus higher during growth on sulfate, than for growth on cysteine. We hypothesize that upon sulfate depletion the high NADPH replenishment causes a surplus of NADPH since no NADPH is used anymore for cysteine assimilation . Phospholipid production, which also requires NAPDH, is then increased as a sink for the NAPDH that is produced in excess. As NADPH is one of the key cofactors in the metabolic network and influenced by many reactions^33^ it can be argued whether a short perturbation causes a prolonged state of OMV release. After sulfur depletion, increased levels of serine could be expected since no sulfide is available anymore to produce cysteine. Besides conversion to cysteine, serine can be used for the production of phospholipids by the conversion to phosphatidylserine. Engineering the redox metabolism is a method for enhanced lipid production in microbes^34^. For example, lipid production in *Yarrowia lipolytica* has been boosted by introduction of pathways that convert NADH to NADPH^35^. Another important role of NADPH is as cofactor of enzymes involved in oxidative stress responses as catalase, superoxide dismutase and glutathione peroxidase^36^. Alternatively, the increased phospholipid production can be required to maintain the membrane integrity because of the increased OMV release. Moreover, sulfur depletion could stimulate OMV release through a secondary mechanism such as oxidative stress. Sulfur is an important component of major oxidative stress responses^37^ and sulfur starvation may induce OMV release by affecting these oxidative stress responses. Oxidative stress responses were observed in the gene expression profile after cysteine depletion previously^24^. As sulfur was unavailable, glutathione biogenesis and iron-sulfur protein biogenesis was impaired. Biologically, Nm encounters sudden oxidative stress in the oxidative burst that phagocytes apply to eliminate invading pathogens^37,38^. Increased OMV release upon oxidative stress can be a method to release oxidative stress or a method to enhance survival upon oxidative bursts.

OMV formation as a result of nutrient limitation has been posed as an evolutionary trigger for OMV release as survival is enhanced through nutrient scavenging^39^. Recently, amino acid deprivation was found to stimulate OMV and tubular OMV structures in *Francisella tularensis*^40^. Nm OMVs have been found associated with iron-scavenging proteins^41^ and *Bacteroides succinogenes* OMVs have been found to contain xylanases or cellulases that can assist in nutrient acquisition^42^. OMV formation upon nutrient limitation may thus assist in nutrient acquisition and improve bacterial survival.

The size of OMVs was similar for OMVs produced after depletion of sulfate and cysteine, while OMV size can be highly subjectable to changes. The induction of OMV release of *Pseudomonas aeruginosa* by gentamycin yields enlarged OMVs^15,43^. Mitomycin C treatment of *Shigella dysenteriae* induces the release of larger OMVs^44^. The size of OMVs can affect both the production process of OMVs, as it influences purification and analysis of OMVs, and the immunogenicity as the size influences the uptake by antigen presenting cells^45^. The observed similarity in size distribution of OMVs in this study is somewhat surprising as the vesicles produced upon sulfate depletion where enriched in phospholipids. This results in a lower content of LPS, which could affect the use of these OMVs as vaccines. The biological activity of native Nm LPS is high, as characterized by its activation of the TLR4/MD-2 complex. LPS can be engineered such, by changes in lipid A acylation and phosphorylation, that these LPS derivates have various strengths of TLR4/MD-2 activation^46^. Future work should tune the required LPS activity of these spontaneously released OMVs with reduced LPS content to optimize the amount of immune activation while limiting toxic side effects for an optimal vaccine composition.

Taken together, the findings of this study expand the knowledge on OMV release by Nm. Sulfur depletion can be used as general mechanism to trigger vesicle formation in Nm and the use of sulfate as sulfur source improves the OMV productivity of Nm batch cultures. Sulfur depletion caused overproduction of phospholipids and especially sulfate depletion resulted in OMVs enriched in phospholipid content. OMV biogenesis remains a complicated and poorly understood process that leaves many research questions remaining. Refined understanding of OMV biogenesis will boost production processes of OMVs released in the supernatant of bacterial cultures and will ultimately make OMV extraction processes obsolete for the production of OMV-based vaccines.

## Materials and Methods

### Shaker flask cultures

A recombinant derivate of the *Neisseria meningitidis* serogroup B isolate H44/76^47^ was used in this study. This strain had a non-encapsulated phenotype due to the *siaD* knockout, *lpxL1* deletion to attenuate LPS-toxicity, *rmpM* deletion to improve vesicle formation and *lgtB* was mutated to skew towards dendritic cells^10,48^. The selected strain was a PorA lacking derivate of the H44/76 isolate. This strain was stored in a two-tiered seedlot system containing glycerol at −135 °C. All cultivations were performed with chemically defined growth medium^49^. Growth on sulfate, sulfite and thiosulfate was performed with adapted strains since cysteine is the preferred sulfur source. Adaptation was performed by subculturing the strain in shaker flasks in medium without cysteine (Supplemental Fig. 1). Initial growth was observed, possibly caused by carryover of cysteine, followed by an adaptation phase of 26 hours. After exponential growth on sulfate was observed, the culture was subcultured on medium with sulfate as the only sulfur source and cryopreserved for further experiments. Shaker flask cultivations of 150 mL were performed in 500 mL baffled shaker flasks incubated at 200 RPM at 35 °C. Cultures were inoculated from a shaker flask culture in the exponential growth phase. Samples with a fixed volume of 2.0 mL were taken for optical density measurements (590 nm), pH measurements and used for sOMV concentration measurements after sterile filtration (0.22 µm) and storage at 4 °C.

### Bioreactor cultures

Batch cultivations were performed in 5 liter dished bottom Applikon bioreactors with an H/D ratio of 1.6 based on total volume. Cultivations with a 3 liter working volume were operated from a Pierre Guerin Tryton^i^ controller. Temperature was controlled at 35 ± 0.5 °C and pH was controlled at pH 7.2 ± 0.05 using 1M HCl and 1M NaOH as titrant. Dissolved oxygen tension (DOT) was controlled at 30%. In the first phase of the cultivation, DOT is controlled by the stirrer (300–1000 RPM) and next the fraction of oxygen is increased in the headspace aeration (1 NL/min). Samples were taken for optical density measurements and used for nutrient and sOMV concentration measurements after sterile filtration (0.22 µm) and storage at 4 °C. Offgas composition was measured by a Thermo Scientific Prima δb process mass spectrometer.

### Proteomics

Protein Digestion: bacterial suspensions were denaturated at 100 °C for 30 min in a potassium phosphate buffer (100 mM, pH 7.8) containing 0.1% Rapigest (Waters) at a protein concentration 0.2 mg/mL. Proteins were digested with 0.25 µg endoproteinase Lys-C (Roche) at 37 °C for 4 h followed by overnight digestion with 1 μg trypsin (Promega) at 37 °C.

Peptide Labeling: relative quantification of proteins in protein samples was performed using dimethyl labeling as described previously^50^. In brief, protein digests were incubated with NaCNBH_3_ and formaldehyde (CH_2_O) in concentrations of 50 mM. The common reference was prepared from 20 μg of each unlabeled digest. The common reference was incubated with NaCNBH_3_ and deuterium-labeled formaldehyde (CD_2_O) in final concentrations of 50 mM. The dimethylated (light) peptide samples were mixed with the deuterium dimethylated (heavy) common reference in a 1:1 molar ratio. Solid phase extraction was used to purify the samples using 1 mL Sep-Pack C18 cartridges and water/acetonitrile/formic acid (40/59.9/0.1%) eluent. Vacuum centrifuge dried peptide mixtures were dissolved in 100 μL water/DMSO/formic acid (94.9/5/0.1%) and diluted 1:20 in water/DMSO/Formic acid (94.9/5/0.1%).

LC−MS/MS Analysis: Nanoscale reversed-phase liquid chromatography electrospray mass spectrometry was used to identify peptides using an Orbitrap Fusion Lumos (Thermo Scientific, USA) and Agilent 1290 (Agilent Technologies, USA), as described previously^51^. Full MS spectra were recorded in the Orbitrap with a resolution of 120 000 (fwhm) (m/z 300−1500) and the 20 most abundant precursors (with a threshold of 25000 counts) were CID fragmented and analyzed in the ion trap. The setpoints of collision energy, isolation width, and isolation offset were set to 35%, 1.6 Da and 0.25 Da respectively. The MD data was processed with Proteome Discoverer 2.2. MS/MS spectra were searched against the *N. meningitidis* H44/76 (NCBI 909420) database with trypsin enzyme specificity and a mass tolerance of 5 ppm for precursor ions and 0.4 for fragment ions. The FDR was set to 1% using the Percolator algorithm (Thermo Scientific). Abundance ratios of the proteins were determined with the default dimethyl quantification workflow of Proteome Discoverer using the dimethyl (C_1_H_3_)_2_ (+28.03130 Da) and (C_1_D_2_H_1_)_2_ (+32.05641 Da) channels.

The protein abundance ratios of the biological triplicates were excluded from the dataset if the protein was detected in less than two out of three replicates. This resulted in a dataset of 621 proteins that were detected in all time-points of the samples. The abundance ratios of the proteins were log2 transformed to obtain fold changes and heat maps were generated.

### OMV purification

From the bioreactor cultures, 50 mL samples were obtained. Samples were centrifuged for 20 minutes at 3000 × *g*, 4 °C, to separate bacteria from the OMV containing supernatant. Next, the supernatant was filtered over a 0.2 µm filter (Nalgene Rapid Flow, PES) and the sterile OMV fraction was concentrated and washed with one volume of 0.01M Tris buffer pH 7.4 containing 3% sucrose on 100 kDa MWCO Amicon Ultra-15 Centrifugal Filter Units according to manufacturer’s protocol (Merck Millipore). Lastly, the OMVs were pelleted by ultracentrifugation (125,000 × *g*, 2 h, 4 °C) and the OMV pellet was resuspended in a 0.01M Tris buffer pH 7.4 containing 3% sucrose.

### OMV composition

Phospholipid and LPS content was analyzed by fatty acid composition measurement using a modified gas chromatography method as described previously^10^. LPS quantification was based on the peak area of C14:3OH (two molecules per LPS moiety) using C12:0-3OH as internal standard. Phospholipid was quantified by the sum of the peak areas C16:0, C16:1, C18:0 and C18:1 using C15:0 as the internal standard.

### Nanoparticle tracking analysis

To analyze purified sOMVs, a NanoSight NS500 (Malvern Instruments) with 488 nm wavelength laser module and sCMOS camera was used for nanoparticle tracking analysis (NTA)^52^. A sample was measured in static mode by capturing 10 movies of 30-seconds with temperature control at 25 °C. Movies were analyzed in the NTA 3.2 software build 3.2.16. The NS500 was calibrated by the NanoSight NTA concentration Measurement upgrade. The sample changer was used to increase the throughput of samples measured^53^. The NS500 is cleaned monthly with Decon 90, following the manufacturers recommendations. Before every set of sample measurements, we confirmed the absence of particles in the MilliQ diluent by measuring the MilliQ diluent for 60 seconds in static mode.

## Supplementary information

Supplementary Figure S1

Supplementary dataset proteomics
