# Supplementary material for: Sulfate depletion triggers overproduction of phospholipids and the release of outer membrane vesicles by Neisseria meningitidis
Source: Sci Rep. 2019 Mar 18;9:4716. doi: 10.1038/s41598-019-41233-x (PMC6423031; doi:10.1038/s41598-019-41233-x)
Supplement: Supplementary file 1 — Supplementary Figure S1 [file 41598_2019_41233_MOESM1_ESM.pdf]

Supplemental Information

Sulfate depletion triggers overproduction of phospholipids and the release of outer membrane vesicles by *Neisseria meningitidis*

Matthias J.H. Gerritzen, Dirk E. Martens, Joost P. Uittenbogaard,
René H. Wijffels, Michiel Stork*

*Correspondence: michiel.stork@intravacc.nl

Supplemental figures

Supplemental Figure S1

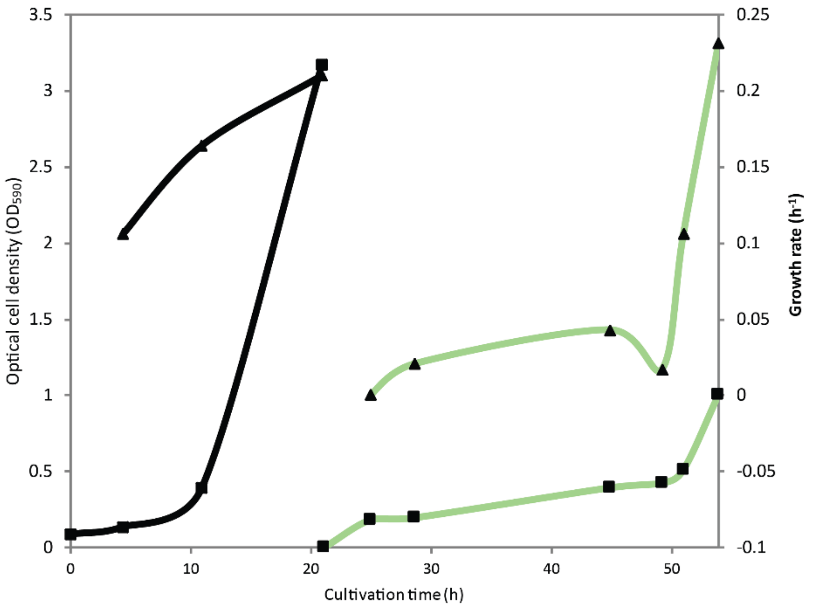

Supplemental Figure 1. Adaptation of *Neisseria meningitidis* from cysteine to sulfate as sulfur source.

Optical density is shown by the square markers, and the growth rate by the triangular markers. Growth on cysteine medium is shown in black and growth on sulfate medium in green. A culture inoculated from a frozen seed was grown on cysteine containing medium (0h - 20h). Next this culture was subcultured to medium with sulfate. A period of adaptation (22h - 48h) was required until growth is observed.
